# Homogenous HIV-1 subtype B from the Brazilian Amazon with infrequent diverse BF1 recombinants, subtypes F1 and C among blood donors

**DOI:** 10.1371/journal.pone.0221151

**Published:** 2019-09-09

**Authors:** Myuki Alfaia Esashika Crispim, Mônica Nogueira da Guarda Reis, Claudia Abrahim, Dagmar Kiesslich, Nelson Fraiji, Gonzalo Bello, Mariane Martins Araújo Stefani

**Affiliations:** 1 Fundação de Hematologia e Hemoterapia do Amazonas/HEMOAM, Amazonas, Manaus, Brazil; 2 Instituto de Patologia Tropical e Saúde Pública, Laboratório de Imunologia da AIDS e da Hanseniase, Universidade Federal de Goiás, Goiânia, Goiás, Brazil; 3 Instituto Oswaldo Cruz, FIOCRUZ, Laboratório de AIDS e Imunologia Molecular, Rio de Janeiro, Rio de Janeiro, Brazil; Centre de Recherche en Cancerologie de Lyon, FRANCE

## Abstract

In the last decade a growing HIV/AIDS epidemic with increased incidence and AIDS-related mortality has been reported in Northern Brazil from which molecular data are scarce. Also, apparently healthy, adult blood donors, recently diagnosed with HIV-1 represent important sentinel populations for molecular studies. This cross-sectional study describes HIV-1 subtypes in blood donors from three reference public blood centers located in three States in Northern Brazil. HIV-1 pol sequencing (protease/PR, reverse transcriptase/RT) was performed on plasma samples of HIV-1 positive donors from HEMOAM, Manaus, Amazonas (*n* = 198), HEMERON, Porto Velho, Rondônia (*n* = 20) and HEMORAIMA, Boa Vista, Roraima (*n* = 9) collected from 2011–2017. HIV-1 subtypes were identified by REGA, phylogenetic inference; recombinant viruses were characterized by SIMPLOT. Young, single, males predominated, around half was first-time donors. Syphilis co-infection was detected in 17% (39 out of 227), 8% (18 out of 227) was anti-HBc positive. Subtype B represented ≥ 90% in Amazonas, Rondônia and Roraima, subtype C (3.1%) was found in Amazonas and Rondônia; subtype F1 (0.9%) and BF1 recombinants (5.3%) were only detected in Amazonas. Subtype B sequences from Amazonas (*n* = 179), Rondônia (*n* = 18) and Roraima (*n* = 9) were combined with viral strains representative of the B_PANDEMIC_ (*n* = 300) and B_CARIBBEAN_/B_CAR_ (*n* = 200) lineages. The B_PANDEMIC_ lineage predominated (78%) although B_CAR_ lineages were frequent in Roraima (56%) and Amazonas (22%). Subtype C and subtype F1 sequences identified here clustered within Brazilian C_BR_ and F1_BR_ lineages, respectively. Twelve BF1 mosaics showed 11 different recombination profiles: six were singleton unique-recombinant-forms/URFs, one displays a CRF28/29_BF-like recombinant pattern and the remaining four BF1 isolates branched with other Brazilian BF1 viruses previously described and may represent putative new CRF_BF1 from Northern Brazil. Our study shows a highly homogeneous molecular pattern with prevalent subtype B, followed by BF1, and sporadic subtype C and F1 in blood donors from the Northern region. Surveillance studies are important to monitor HIV-1 diversity which can reveal patterns of viral dissemination, especially in a highly endemic, remote and geographically isolated region as Northern Brazil.

## Introduction

Brazil is a vast and diverse country and the most affected by the HIV/AIDS epidemic in the Americas. The Brazilian AIDS epidemic is very heterogeneous reflecting the great socio-cultural and economic disparities seen in the country. From 2006 to 2016 the national AIDS incidence rate decreased 9.4%, however, during the same period a significant increase (42.2%) was reported in the Northern region that comprises seven States, three of them (Amazonas, Roraima and Rondônia) were investigated in this study [1]. In 2017, the Roraima state reported the highest AIDS incidence in the country, with a 36.8% increase in the last decade, while the Amazonas state ranked fourth with 29.6% growth [1]. Between 2007–2017, AIDS related mortality rate fell 14.8% in Brazil, but augmented significantly in most States from the Northern region [1]. These official data highlight the crucial importance of surveillance studies in Northern Brazil, which is characterized by an enormous territorial area, remote from the most populated and industrialized regions and with low population density.

The molecular epidemiology of HIV-1 in Brazil is complex and dynamic and has been characterized by the co-circulation of several “pure” group M subtypes. Studies from distinct Brazilian regions have described the preponderance of subtype B, except in the Southern region where subtype C predominates [2–12]. Also a previous study reported that the HIV-1 subtype B pandemic lineage (B_PANDEMIC_) is prevalent compared to the Caribbean non-pandemic subtype B clades (B_CAR_), except in some states of Northern Brazil [13]. Recent Brazilian studies have reported a growing number of non-subtype B infections, mainly subtype C, while subtype F1 remains sporadic except in the Northeastern [14,15]. Also, an escalating contribution of BF1 and BC hybrids classified as circulating recombinant forms (CRFs) or unique recombinant forms (URFs) have been described [14,16,17,18]. Data on the epidemiological and molecular features of this expanding AIDS epidemic in Northern Brazil are still scarce [17,19–27]. Mapping of HIV-1 genetic diversity can reveal the patterns of viral dissemination especially in a highly endemic, remote and geographically isolated region as Northern Brazil. A previous historical series from 1992–2012 at HEMOAM, the only public reference hemocenter in Amazonas state showed a significant number of HIV-1 infections among blood donors [28]. In this context, apparently healthy, adult blood donors recently diagnosed with HIV-1 infection represent important sentinel populations for molecular studies. This study characterized HIV-1 genetic diversity in recently diagnosed blood donors from three reference public blood centers located in Northern Brazil.

## Material and methods

### Study area and study population

**Fig 1:**
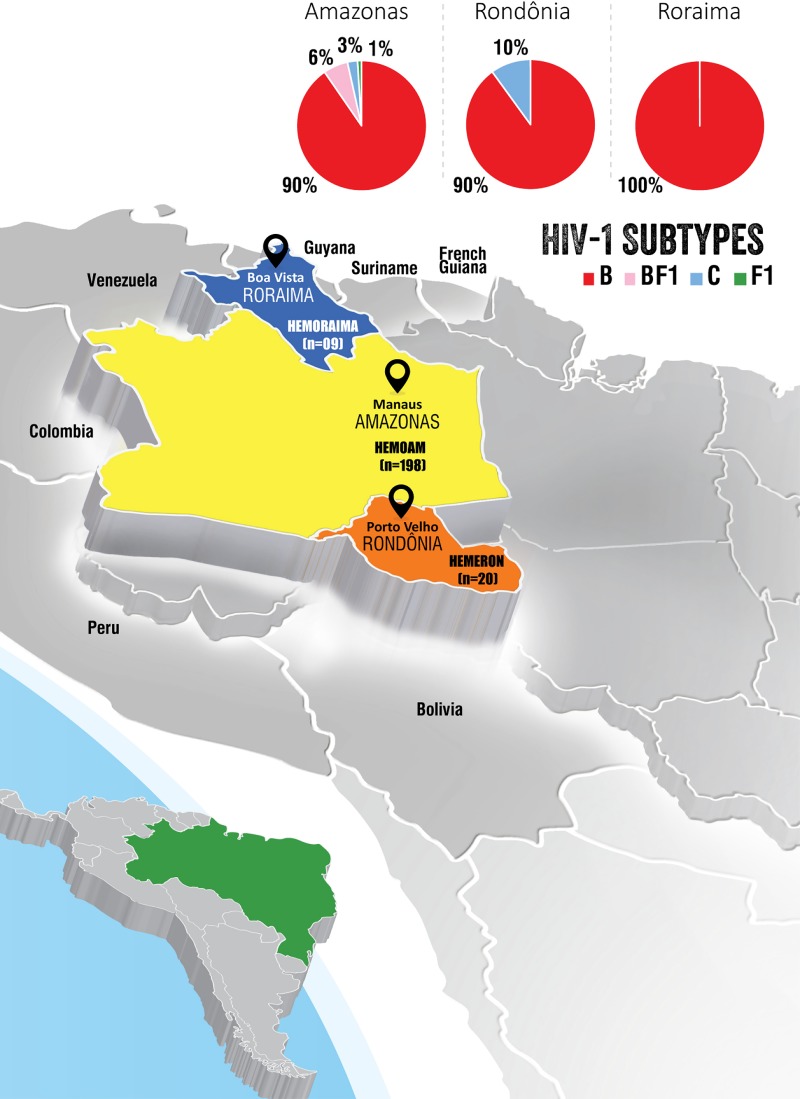
Map of Latin America, Brazil depicting our study area in the Northern Brazilian states of Amazonas, Rondônia and Roraima. The participating blood centers are highlighted: HEMOAM in Manaus, Amazonas, HEMERON in Porto Velho, Rondônia and HEMORAIMA in Boa Vista, Roraima. The proportions of the HIV-1 subtypes B, F1, C and BF1 recombinants identified in each hemocenter are shown in the pie-chart.

This is a cross sectional study in convenience samples collected from 2011–2017 among blood donors with diagnosis of HIV-1 infection in three blood centers located in the states of Amazonas (AM), Rondônia (RO) and Roraima (RR) in Northern Brazil (Fig 1). The following reference public blood centers participated in this study: Fundação de Hematologia e Hemoterapia do Amazonas/HEMOAM, Manaus, AM, Fundação Hematologia e Hemoterapia de Rondônia/HEMERON, Porto Velho, RO, and Hemocentro de Roraima/HEMORAIMA, Boa Vista, RR. The HEMOAM is located in Manaus, the capital of Amazonas (4 million inhabitants, area: 1.571,000 km^2^, population density: 2.23 inhabitants/km^2^), the HEMERON is located in Porto Velho, capital of Rondônia (1.8 million inhabitants, area: 237.765,293 km^2^, population density: 6.58 inhabitants/km^2^) and HEMORAIMA is situated in Boa vista, capital of Roraima which is the least populated Brazilian state (522,636 inhabitants, area: 224.300,805 km^2^, population density: 2.01 inhabitants/km^2^) [29] (Fig 1). The inclusion criteria comprised recently diagnosed blood donors with HIV-1 infection from any gender, age, donor type (first time and repeat), and donation category (voluntary and replacement). Confirmatory tests included Western blot (HIV-1 BLOT 2.2 (MP Biomedicals SAS, France) and a multiplex real time duplex HIV/HCV PCR nucleic acid test/NAT (Kit NAT HIV/HCV), Bio-Manguinhos, Rio de Janeiro). At the time this study was conducted, samples of blood donors from Rondônia and Roraima testing positive for HIV-1/2 during screening were sent to HEMOAM, Manaus to be tested by the HIV/HCV NAT and the confirmed cases were included in our study sample. As part of the standardized procedure, all donors with a positive result during the serological screening are invited to return to the blood bank to donate another blood sample to be retested. All confirmed cases are invited to return to the blood bank where they are informed about their positive results and instructed to proceed to the local ambulatory service (SAE) from which they are oriented to go to the local referral diagnostic and treatment centers (Manaus/Amazonas: Fundação de Medicina Tropical Dr Heitor Vieira Dourado; Porto Velho/ Rondônia: Hospital de Medicina Tropical; Boa Vista/Roraima: Hospital Coronel Mota).

### Genetic analysis

Plasma samples from blood donors were used for RNA extraction (QIAamp Viral RNA Mini Kit, Qiagen, Hilden, Germany), RNA was reverse transcribed into complementary DNA (cDNA) (Invitrogen) and used as the target for nested polymerase chain reaction (nested-PCR). The HIV-1 protease (PR) and reverse-transcriptase (RT) K1/K2 external primers and DP10/F2 internal primers [30,31] amplify the entire PR region (positions 2253–2549 relative to HXB2 genome, GenBank accession no. K03455) and a 750 bp fragment of RT region (positions 2550–3299 relative to HXB2 genome). Amplicons were purified (QIAquick PCR Purification Kit/QIAGEN, Qiagen GmbH, Hilden, Germany) and genomic sequencing was performed (Big Dye Terminator Sequencing kit v. 3.1, Applied Biosystems, USA; ABI Prism 3100 Genetic Analyzer, Applied Biosystems, USA). All generated sequences were subjected to quality control analysis by HIV-1 Quality Analysis Pipeline Tool (http://www.sanbi.ac.za) and were screened by visual inspection of the alignment (Bioedit software) to check for sample mix-ups and contamination [32,33].

### Phylogenetic analyses

HIV-1 genetic subtype was defined by REGA automated genotyping tool (version 2.0) and by phylogenetic inference [34,35]. Study sequences were aligned with reference set from the Los Alamos HIV database (ww.hiv.lanl.gov) using the Clustal X software and adjusted manually by the BioEdit software [33,36]. Phylogenetic inferences were performed by the Neighbor-Joining (NJ) method under Kimura’s two-parameter correction using MEGA6 software [37,38]. Bootstrap values (1000 replicates) above 70% were considered significant. Isolates with discordant PR/RT subtypes were analyzed by SIMPLOT 3.5.1 software (200 bp sliding window, advanced in 20 bp steps size increments, 1000 replicates) [39]. Sequences were gap-stripped, the transversion-to-transition ratio was set to 2.0, and distances were calculated according to Kimura’s two-parameter mode. The clustering pattern of Northern Brazilian sequences analyzed here and reference sequences was investigated by performing maximum-likelihood (ML) phylogenetic analyses. HIV-1 subtype B *pol* sequences from Northern Brazil were aligned with subtype B references sequences representative of the B_PANDEMIC_ clade and major B_CAR_ clades circulating in the Caribbean and South America described previously [13,40–42]. The HIV-1 subtype F1 *pol* sequences from Northern Brazil were aligned with subtype F1 references sequences representative of Brazilian (F1_BR_), Romanian and Central African clades described previously [43]. The HIV-1 subtype C *pol* sequences from Northern Brazil were aligned with subtype C references sequences representative of Brazilian (C_BR_), Eastern African, Southern African and Central African clades described previously [44,45] The BF1 recombinants sequences here identified were aligned with all URFs_BF1 and CRFs_BF1 of Brazilian origin available at Los Alamos HIV Database. ML phylogenetic trees were reconstructed with the PhyML 3.0 program [46] under the best nucleotide substitution model, selected by the SMS (Smart Model Selection) software [47] integrated into the PhyML web server. The SPR branch-swapping algorithm was selected for heuristic tree search and the approximate likelihood-ratio test (a*LRT*) [48] to estimate the reliability of the tree topology obtained. The GenBank accession numbers of the sequences presented in this study are MH673055-MH673281.

### Data analysis

Frequencies and medians were calculated and the *x*^2^ test used for categorical variables and the Mann Whitney test for continuous variables.

### Ethics issues

The ethical issues of this study were reviewed by the institutional IRB (“Comitê de Ética em Pesquisa com Seres Humanos, Fundação de Hematologia e Hemoterapia do Amazonas”, protocol # 31061814.6.0000.0009), which approved this study and waived the requirement for informed consents.

## Results

### Main socio-demographic and donation characteristics of HIV-1 infected blood donors from the Brazilian Amazon

Our study population consisted of 227 blood donors recently diagnosed with HIV-1 infection between 2011–2017 in three reference public blood centers from Northern Brazil (Fig 1 and Table 1). Most participants were from HEMOAM, AM (*n* = 198, 87%), twenty (9%) were from HEMERON, Rondônia and nine (4%) were from HEMORAIMA, Roraima. The majority of participants in these blood centers was male (88%, 200 out of 227) between 20–40 years (82%, 185 out of 227) (Table 1). Unmarried individuals represented 78% (177 out of 227) and the smallest prevalence of singles was seen in Rondônia (55%, 11 out 20). First time and repeat donors were reported at comparable rates with similar profiles in Amazonas and Rondônia, while in Roraima repeat donors predominated (Table 1). The majority of participants (70%, 158 out of 227) reported up to 11 schooling years but in Rondônia donors with this educational level represented (50%, 10 out 20). Blue-collar workers were 60% (137 out of 227) with similar rates in all three centers. Syphilis co-infection was detected in 17% (39 out of 227) and 8% (18 out of 227) was anti-HBc positive, suggesting previous or ongoing infection with HBV, with predominance of these markers at HEMOAM, Amazonas.

**Table 1:** Main socio demographic and donation characteristics of HIV-1 infected blood donors from Amazonas, Rondônia and Roraima states in the Brazilian Amazon.

Characteristics	Total	Amazonas	Rondônia	Roraima
	n = 227 (%)	n = 198 (%)	n = 20 (%)	n = 09 (%)
**Gender**				
Male	200 (88.1)	177 (89.4)	15 (75.0)	8 (88.9)
Female	27 (11.9)	21 (10.6)	5 (25.0)	1 (11.1)
**Age Range (years)**				
18–20	8 (3.5)	7 (3.5)	1 (5.0)	
20–40	185 (81.5)	162 (81.9)	16 (80.0)	7 (77.8)
40–65	34 (15.0)	29 (14.6)	3 (15.0)	2 (22.2)
**Marital Status**				
Single	177 (78.0)	157 (79.3)	11 (55.0)	9 (10)
Married	50 (22.0)	41 (20.7)	9 (45.0)	
**Type of Donor**				
First time	115 (50.7)	101 (51.0)	10 (50.0)	4 (44.4)
Repeat	112 (49.3)	97 (49.0)	10 (50.0)	5 (55.6)
**Type of Donation**				
Replacement	114 (50.2)	91 (46.0)	17 (85.0)	6 (66.7)
Voluntary	113 (49.8)	107 (54.0)	3 (15.0)	3 (33.3)
**Education level**				
≤8 years	26 (11.4)	23 (11.6)	2 (10.0)	1 (11.1)
≤11 years	158 (69.6)	142 (71.7)	10 (50.0)	6 (66.7)
university	24 (10.6)	22 (11.1)		2 (22.2)
NA	19 (8.4)	11 (5.6)	8 (40.0)	
**Occupation**				
Student	28 (12.3)	20 (10.1)	6 (30.0)	2 (22.2)
Military	20 (8.8)	20 (10.1)		
Blue-collar worker	137 (60.4)	119 (60.1)	13 (65.0)	5 (55.6)
Others	42 (18.5)	39 (19.7)	1 (5.0)	2 (22.2)
**Co-infection**				
anti-HBc	18 (7.9)	18 (9.1)		
Syphilis	39 (17.2)	38 (19.2)		1 (11.1)

### HIV-1 genetic diversity

HIV-1 subtype B was the major genetic variant in blood donors from all three hemocenters (91%; 206 out of 227), ranging from 90% in Amazonas and Rondônia to 100% in Roraima (Fig 1). Overall, subtype C represented 3.1% (7 out of 227), ranging from 0% in Roraima to 10% in Rondônia. Subtype F1 was 1% (2 out of 227) and the prevalence of BF1 recombinants was 5% (12 out of 227). Subtype F1 and BF1 recombinants were only identified in Amazonas state (Fig 1 and Table 1).

### Phylogenetic analyses of HIV-1 subtype B sequences

**Fig 2:**
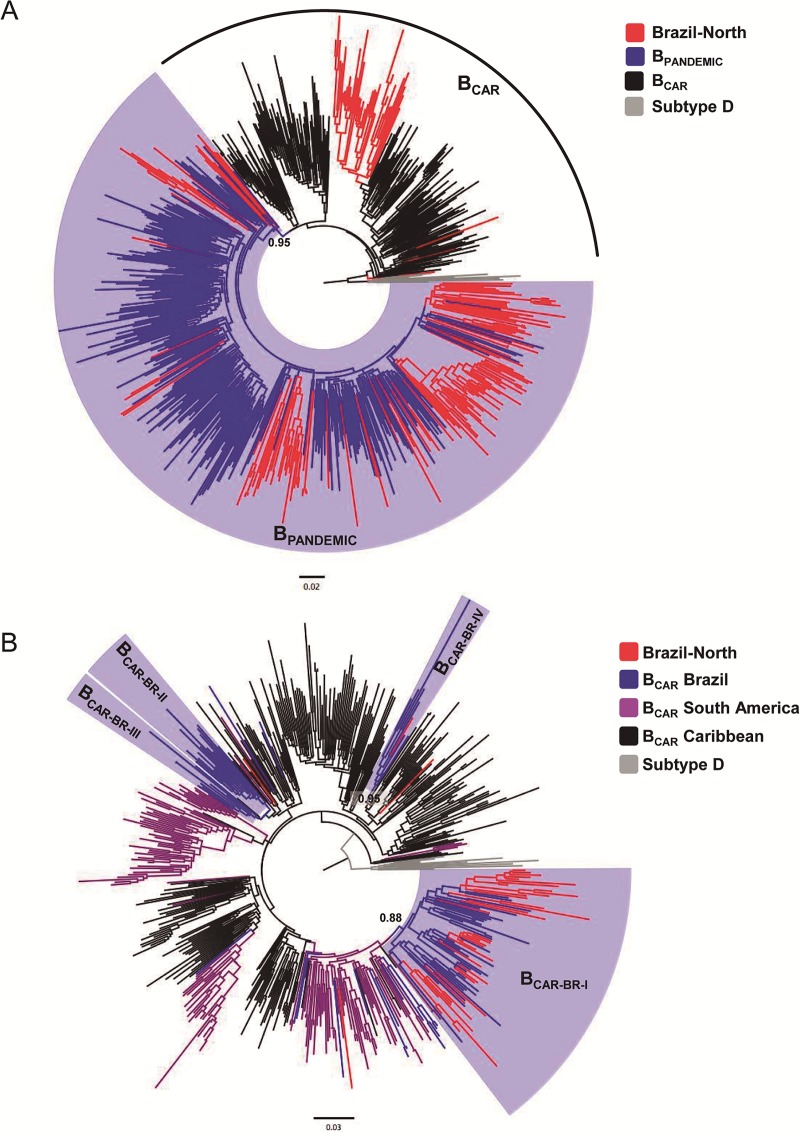
Maximum-likelihood (ML) phylogenetic analyses of HIV-1 subtype B_PANDEMIC_ and B_CAR_ strains identified in Northern Brazil. (A): A total of 206 HIV-1 subtype B sequences from this study (179 from Amazonas, 18 from Rondônia, 9 from Roraima, depicted in red) was combined with 300 previously described viral strains representative of the B_PANDEMIC_ (depicted in blue) and 200 B_CAR_ lineages (black color). (B): The 45 B_CAR_ sequences identified in blood donors from Amazonas, Rondônia and Roraima (depicted in red) were combined with previously identified viral strains representative of the major B_CAR_ lineages circulating in Brazil (blue color), Northern South America (French Guiana and Suriname) (purple color) and the Caribbean (Hispaniola, Jamaica and Trinidad and Tobago) (black color).

HIV-1 subtype B sequences from Amazonas (*n* = 179), Rondônia (*n* = 18) and Roraima (*n* = 9) here identified were combined with viral strains representative of the B_PANDEMIC_ (*n* = 300) and B_CAR_ (*n* = 200) lineages. The ML phylogenetic analysis revealed that B_PANDEMIC_ was 78% and B_CAR_ was 22%. A substantial proportion of subtype B sequences from Roraima (56%) and Amazonas (22%) and a low percentage of sequences from Rondônia (6%) were intermixed among basal B_CAR_ strains, while remaining sequences branched with high support (a*LRT* = 0.95) within the B_PANDEMIC_ clade (Fig 2A and Table 2,). The B_CAR_ sequences from Amazonas, Rondônia and Roraima (*n* = 45) were then combined with previously identified viral strains representative of the major B_CAR_ lineages circulating in the Caribbean (Hispaniola, Jamaica and Trinidad and Tobago), Northern South America (French Guiana and Suriname) and Brazil. This new ML phylogenetic analysis revealed that most B_CAR_ from Northern Brazil (91%) branched (a*LRT* = 0.88) within the major Brazilian B_CAR-BR-I_ clade, characteristic of this region. Two sequences from Roraima branched (a*LRT* = 0.95) within the Brazilian clade B_CAR-BR-IV_ (together with other sequences from the same Brazilian state) and a few proportion of sequences (7%) branched as sporadic lineages intermixed among sequences from the Caribbean and Northern South America (Fig 2B and Table 3).

**Table 2:** Subtype B clade assignment: B_PANDEMIC_ and B_CARIBBEAN_/ B_CAR_.

Brazilian State	Subtype B (total)	B_PANDEMIC_ (N, %)	B_CAR_ (N, %)
Amazonas	179	140 (78%)	39 (22%)
Rondônia	18	17 (94%)	1 (6%)
Roraima	9	4 (44%)	5 (56%)
Total	206	161 (78%)	45 (22%)

**Table 3:** Subtype B_CAR_ sub-clade assignment.

Brazilian State	B_CAR_	B_CAR-BR-I_	B_CAR-BR-IV_	Sporadic Lineages
Amazonas	39	36 (92%)	-	3 (8%)
Rondônia	1	1 (100%)	-	-
Roraima	5	4 (80%)	1 (20%)	-
Total	45	41 (91%)	1 (2%)	3 (7%)

### Phylogenetic analyses of HIV-1 subtype C and F1 sequences

**Fig 3:**
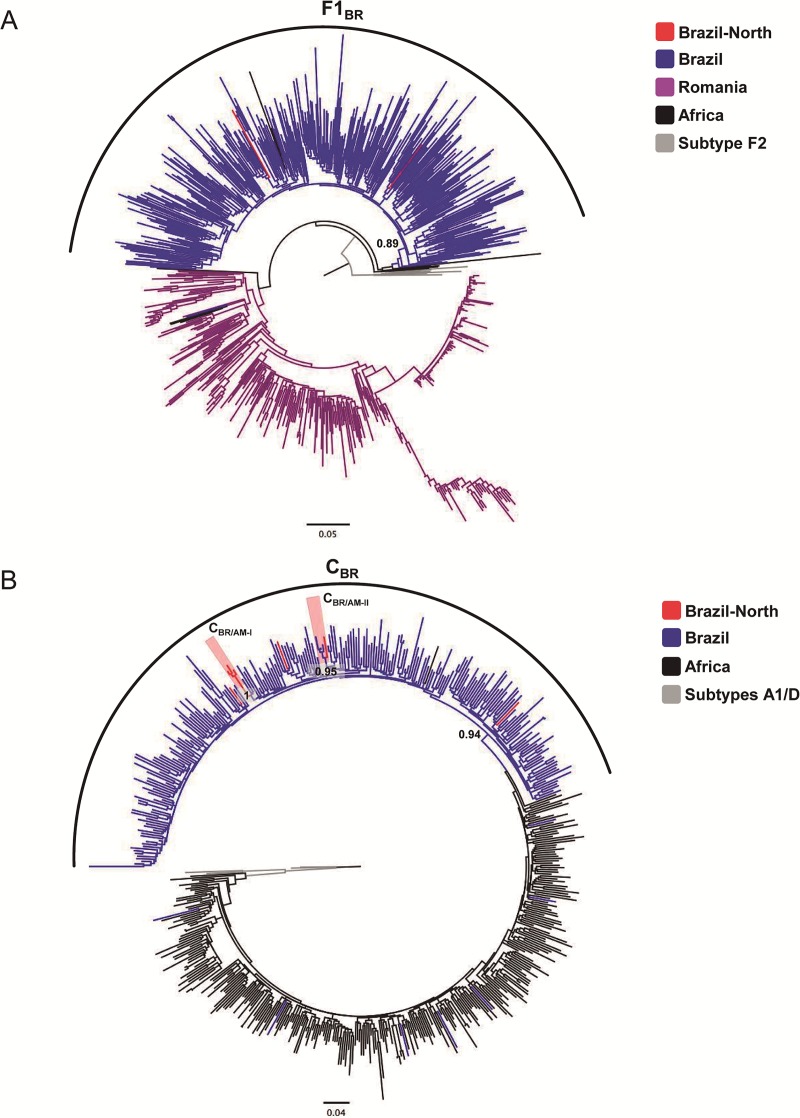
**A. Maximum-likelihood phylogenetic analyses of HIV-1 subtype F1 *pol* sequences identified in blood donors from Northern Brazil.** Subtype F1 sequences described in this study (depicted in red) were aligned with previously described subtype F1 references sequences representative of Brazilian (F1_BR_, depicted in blue) Romanian (purple) and Central African clades (black). **B. Maximum-likelihood phylogenetic analyses of HIV-1 subtype C *pol* sequences detected in blood donors from Northern Brazil.** Subtype C sequences described in this study (depicted in red) were aligned with previously described subtype C references sequences representative of Brazilian (C_BR_, blue), Eastern African, Southern African and Central African clades (black).

In order to better understand the putative origin of the subtype C sequences detected in the Amazonas (*n* = 5) and Rondônia (*n* = 2) states, these sequences were aligned with sequences representative of major regional lineages already described in Brazil (C_BR_), and Central, Southern and Eastern Africa. Similarly, subtype F1 sequences detected in the Amazonas state (*n* = 2) were aligned with sequences representative of major lineages circulating in Brazil (F1_BR_), Europe (Romania) and Central Africa. The ML analyses revealed that all subtypes F1 and C sequences detected in Amazonas and Rondônia branched with high support within the F1_BR_ (a*LRT* = 0.89, Fig 3A) and the C_BR_ (a*LRT* = 0.94, Fig 3B) Brazilian clades. The two subtype F1 sequences identified branched in separate branches together with sequences from other Brazilian states. Among the seven subtype C sequences identified, three branched as independent lineages together with sequences from Southern Brazilian states. The remaining four subtype C sequences branched in two highly supported (a*LRT* ≥ 0.95) monophyletic clusters only comprising sequences from Amazonas state (Fig 3B). The lineage called C_BR/AM-I_ comprises three sequences here detected, while the lineage C_BR/AM-II_ comprises one sequence here detected and three sequences detected in a previous study [17].

### Phylogenetic analyses of HIV-1 BF1 recombinant sequences

**Fig 4:**
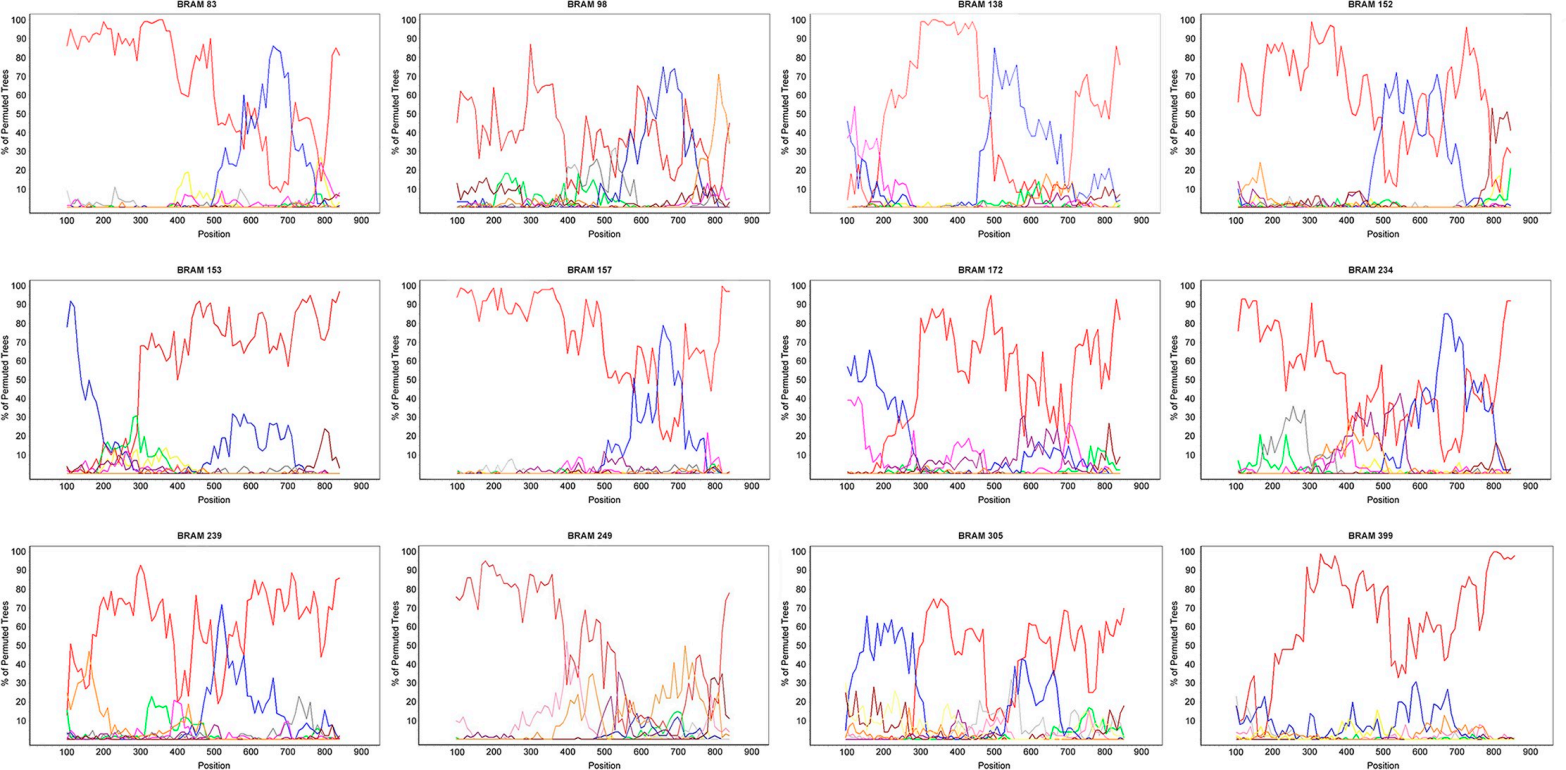
Bootscanning analyses of HIV-1 pol sequences of 12 BF1 recombinant viruses detected in blood donors from Northern Brazil. HIV-1 subtypes are indicated by different colors, Subtype A: yellow, Subtype B: red, Subtype C: green, Subtype D: pink, Subtype F1: blue, Subtype G: purple, Subtype H: gray, Subtype J: brown, Subtype K: orange. SIMPLOT 3.5.1 software was employed and recombination breakpoints were estimated using 200 bp sliding window, advanced in 20 bp steps size increments, 1000 replicates.

**Fig 5:**
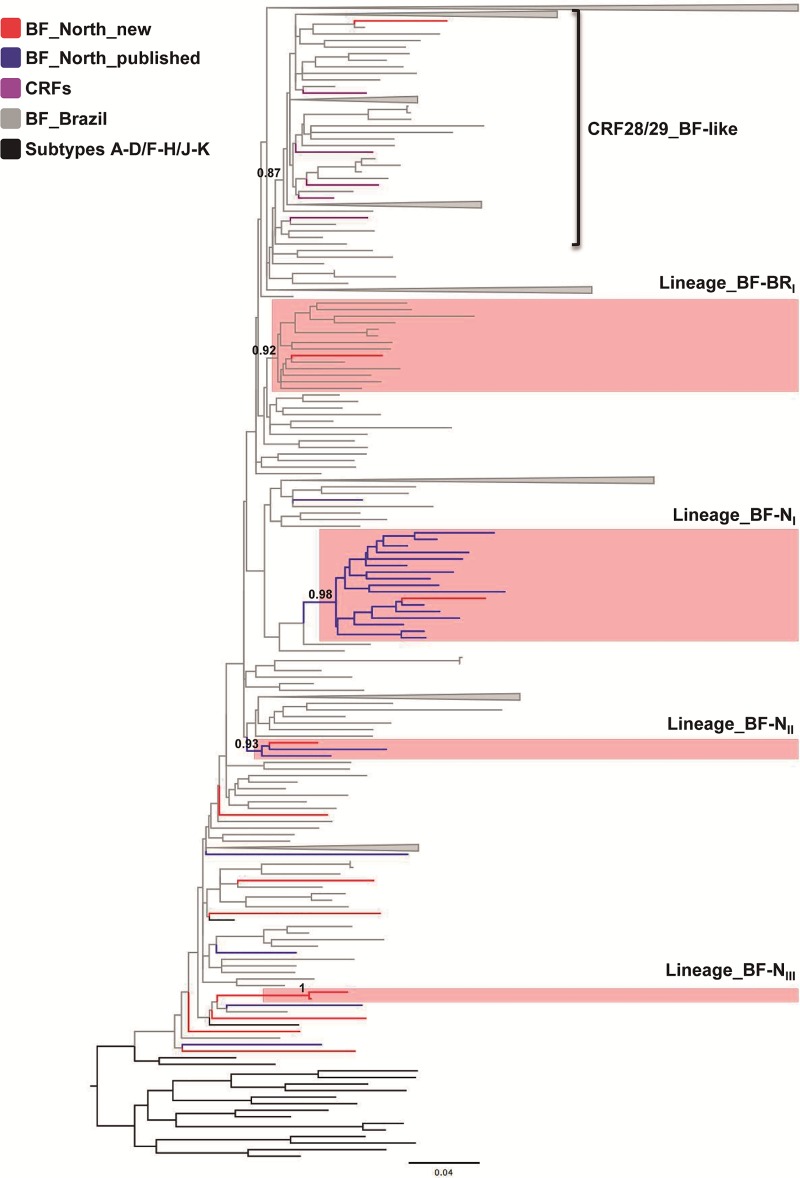
Maximum-likelihood phylogenetic analyses of 12 BF1 recombinant sequences from the Northern region. BF1 here identified (depicted in red) are shown together with all Brazilian BF unique recombinant forms/URFs and BF circulating recombinant forms/CRF sequences available in the Los Alamos HIV Database. CRF28_BF and CRF29_BF like lineages were identified and BF strains that originated the monophyletic lineages denoted as BF-BR_I_, BF-N_I_, BF-N_II_ and BF-N_III_ are highlighted in pink.

In this study 12 BF1 recombinant sequences with 11 different recombination profiles were identified in Amazonas state (Fig 4). The estimated recombination points of BRAM_83 and BRAM_157 were similar (Fig 4). All BF1 recombinants sequences from the Northern region here identified were combined with all Brazilian URFs/CRFs sequences available in the Los Alamos HIV Database. The ML analysis showed that the 12 BF1 recombinant sequences here detected were distributed in 11 independent lineages as BRAM_83 and BRAM_157 isolates branched together (Fig 5). Six BF1 sequences did not branch with high support with any other Brazilian URFs/CRFs sequences and were thus classified as URFs. One BF1 sequence branched (a*LRT* = 0.87) together with CRF28/29_BF reference sequences, being thus classified as CRF28/29_BF-like recombinant (Fig 5). The remaining four BF1 sequences branched with high support (a*LRT* ≥ 0.87) with other Brazilian URFs originating the monophyletic lineages here called BF-BR_I_, BF-N_I_, BF-N_II_ and BF-N_III_ (Fig 5). The lineage BF-BR_I_ comprises 14 BF1 sequence (Fig 5), including one from Amazonas, eight from São Paulo and five from unknown Brazilian state origin (Fig 5). The lineage BF-N_I_ includes 17 BF1 sequences from Amazonas (*n* = 8), Rondônia (*n* = 5), Roraima (*n* = 3) and Acre (*n* = 1) states and was described previously as a putative new CRF_BF characteristic of the Northern Brazilian region [17]. The lineages BF-N_II_ and BF-N_III_ consist of three and two BF1 sequences from Amazonas state, respectively (Fig 5).

## Discussion

Our molecular study with HIV-1 isolates of recently diagnosed blood donors from three public reference blood centers from Northern Brazil showed a marked predominance of subtype B (≥90%), both B_PANDEMIC_ and B_CAR_ lineages. Following the introduction of a founder subtype B strain from Central Africa into Haiti around the middle 1960s, subtype B became a major variant in the Americas and in all Brazilian regions, except for the Southern region [2–12]. Our results corroborate previous reports which showed the dominance of subtype B (81%) among 305 HIV-1 *pol* sequences from ARV-exposed patients in five states from Northern Brazil ranging from 63% in Rondônia to 92% in Acre [17]. In 97 ARV naïve individuals from Amapá state, subtype B represented 74% [24]. Subtype B was also highly prevalent in Pará state representing 97% of 34 protease sequences of pregnant women [25] and 85% in patients failing HAART [22]. A previous study in 31 HIV-1 sequences of blood donors from Amazonas also showed the predominance of subtype B [21] and a recent study in 129 antiretroviral naïve children from Manaus showed 80.2% subtype B infections [27]. Only subtype B was identified in Boa Vista, the capital of Roraima, the northern most Brazilian state, which is only connected by road to Amazonas state, Venezuela and Guyana. Despite the small sample size from Roraima, our findings are in accordance with a recent study in 73 patients that showed 91% subtype B infections in this state [26]. Our results show that the highly uniform subtype B epidemic described in Northern Brazil is similar to the epidemic reported in bordering countries as Venezuela, French Guiana and Suriname [49–51]. Also, the subtype B prevalence here detected in blood donors from the Northern Brazil is higher than that previously described (<70%) in blood donors from the Southeastern and Northeastern regions [14, 21]. A recent report by our group described 11% transmitted drug resistance/TDR rate (25 out of 227) in these donors: 10.1% non-nucleoside-reverse-transcriptase-inhibitor/NNRTI mutations, 5.3% nucleoside-reverse-transcriptase-inhibitor/NRTI mutations and 0.4% protease-inhibitor/PI mutations; NNRTI/NRTI mutations represented 4.8%. Also, three highly supported subtype B monophyletic clades mostly composed by individuals from Amazonas with TDR/drug resistance mutations were identified suggesting transmission clusters of multi-drug-resistant viruses in these blood donors [52].

While other continental American countries show the predominance of the globally disseminated B_PANDEMIC_ lineage, our results confirm a high prevalence of Caribbean non-pandemic subtype B_CAR_ lineages in Amazonas and Roraima states [13]. The subtype B epidemic in the bordering countries of French Guiana and Suriname is characterized by the B_PANDEMIC_ and the B_CAR_ lineages with multiple active transmission chains of both lineages [42,51]. A similar mixed pattern of B_PANDEMIC_ and B_CAR_ lineages was reported in several Caribbean islands [41]. After 2010 the Northern Brazilian states have received substantial migration waves from Haiti, located in the Caribbean Sea, triggered by the collapse in the local infrastructure caused by the earthquake [53]. Most B_CAR_ strains from Northern Brazil, however, do not branch among B_CAR_ sequences from the Caribbean, but within the major B_CAR-BR-I_ clade that probably arose in the Northern Brazilian region around the late 1970s [13]. This clearly supports that most B_CAR_ infections detected in Northern Brazil resulted from the expansion of long-standing local transmission networks and not from recent introductions of B_CAR_ strains from the Caribbean region.

Our results from northern Brazil confirm the growing role and the complexity of non-B subtype variants in the AIDS epidemic throughout the country. BF1 recombinants were the second most prevalent variant among blood donors from Amazonas, although identified at a much lower rate than found in other geographic regions (5.3% in AM versus 11% in São Paulo and 29% in different regions) [14, 21]. In our study, a remarkable genetic diversity was found in the 12 BF1 recombinants that showed 11 different recombination profiles compatible with new URFs BF1, CRF28/29_BF-like and two putative new CRFs BF1, one of them exclusive of Northern Brazil. All of the twelve patients harboring BF1 recombinant viruses were originally from North Brazil: three were from Pará State and nine from Amazonas state, suggesting the local generation of these BF1 recombinant forms. Also, our analyses with all other BF1 recombinants from Brazil suggested a putative new CRF_BF from Northern Brazil. Nevertheless, our results did not indicate any new CRF_BF circulating among investigated blood donors. Previous studies in blood donors described the CRF70_BF1 and the CRF71_71 in near full-length proviral genomes of blood donors from Pernambuco, Northeastern Brazil [54] and the CRF72_BF1 was identified in five blood donors from Minas Gerais, Southeastern Brazil [55].

In the North region subtype C was first described in 2012 among blood donors from Amazonas and it has been shown to circulate at low proportions in other states from the North region ranging from 1% in Amapá to 9% in Tocantins [5, 17, 23–26]. Subtype C epidemic was introduced by a single founder strain from East Africa into Southern Brazil and from there it has disseminated throughout the country originating the major C_BR_ clade [43]. Subtype C sequences identified here belong to the major C_BR_ Brazilian lineages, confirming that dissemination of subtype C northwards probably reflects migration of southern individuals for deforestation, agricultural and cattle activities in north Brazil. In fact, among the three patients harboring subtype C infections, two were originally from the Southeast and one was from the South region suggesting that these subtype C infections detected in North Brazil were imported cases. Nevertheless, our study shows for the first time, two local transmission clusters of subtype C in Amazonas state. Subtype F1 has been reported as a minor variant in most Brazilian regions, except in Pernambuco where significant prevalence was found [7,15]. Subtype F1 has been previously reported to be prevalent in Amazonas in early 2000 [19], however further studies have detected only sporadic cases of subtype F1, similarly to our findings [21,22]. In Brazil full genome sequence analyses of isolates originally classified as subtype F1 in the pol region revealed that a significant proportion was in fact BF1 recombinants [56]. Further HIV-1 full-length genomic studies are necessary to characterize putative new CRFs-BF and to define the real proportion of “pure” subtype F1 circulating in Northern Brazil.

Our study has some potential limitations such as the small number of participants from Roraima, however molecular data from this state is very scarce with a limited number of antiretroviral-naïve sequences from this state available at the GenBank. Information on CD4 counts and viral loads of recently diagnosed blood donors were not available and these data could define better the immunological and virological profiles of these individuals. We have used a convenience sample that contained the great majority of cases, however it does not reflect the actual profile of all HIV positive donors limiting calculations about incident cases and about the frequency of co-infections, particularly syphilis and HBsAg among donors. Also the apparent limited genetic diversity may have been also underestimated, as it does not exclude the possibility of identification of other subtypes in other genomic regions that were not evaluated. However, one advantage of our study was the use of residual plasma samples from donations, which eliminates the bias associated to donor return.

In conclusion, our in-depth molecular data about HIV-1 epidemic in recently diagnosed blood donors from public reference centers in three states, Northern Brazil confirm a marked predominant subtype B epidemic with mixed B_PANDEMIC_ and B_CAR_ lineages. Also highly diverse BF1 recombinant strains and a smaller contribution of subtypes F1 and C infections were found. Recently, intense human flux across the Northern Brazilian border to escape from politic, economic and humanitarian crisis in Haiti and Venezuela, overwhelms the public healthcare infrastructure in in this region and has been promoting poverty, malnutrition, commercial sex and dissemination of infectious diseases in these vulnerable and under-immunized communities. Population growth and human migration and mobility are recognized to play an important role in shaping the HIV/AIDS epidemic [57]. In this context, molecular epidemiologic surveillance studies will be key to monitor spatiotemporal changes in HIV-1 diversity in the highly endemic Northern region.
